# Designing of a Recombinant Multi-Epitopes Based Vaccine against *Enterococcus mundtii* Using Bioinformatics and Immunoinformatics Approaches

**DOI:** 10.3390/ijerph19063729

**Published:** 2022-03-21

**Authors:** Metab Alharbi, Abdulrahman Alshammari, Abdullah F. Alasmari, Salman Mansour Alharbi, Muhammad Tahir ul Qamar, Asad Ullah, Sajjad Ahmad, Muhammad Irfan, Atif Ali Khan Khalil

**Affiliations:** 1 Department of Pharmacology and Toxicology, College of Pharmacy, King Saud University, P.O. Box 2455, Riyadh 11451, Saudi Arabia; mesalharbi@ksu.edu.sa (M.A.); abdalshammari@ksu.edu.sa (A.A.); afalasmari@ksu.edu.sa (A.F.A.); 2 Ministry of Health, Kingdom of Saudi Arabia, Riyadh 11451, Saudi Arabia; salman20-13@hotmail.com; 3 Department of Bioinformatics and Biotechnology, Government College University, Faisalabad 38000, Pakistan; 4 Department of Health and Biological Sciences, Abasyn University, Peshawar 25000, Pakistan; asadullahaup@gmail.com; 5 Department of Oral Biology, College of Dentistry, University of Florida, Gainesville, FL 32611, USA; irfanmuhammad@ufl.edu; 6 Department of Biological Sciences, National University of Medical Sciences, Rawalpindi 46000, Pakistan; atif.ali@numspak.edu.pk

**Keywords:** antibiotic resistant, *Enterococcus mundtii*, subtractive proteomics, molecular docking, molecular dynamic simulation

## Abstract

Enterococcus species are an emerging group of bacterial pathogens that have a significant role in hospital-associated infections and are associated with higher mortality and morbidity rates. Among these pathogens, *Enterococcus mundtii* is one of the causative agents of multiple hospital associated infections. Currently, no commercially available licensed vaccine is present, and multi-drug resistant strains of the pathogen are prominent. Due to several limitations of experimental vaccinology, computational vaccine designing proved to be helpful in vaccine designing against several bacterial pathogens. Herein, we designed a multi-epitope-based vaccine against *E. mundtii* using in silico approaches. After an in-depth analysis of the core genome, three probable antigenic proteins (lytic polysaccharide monooxygenase, siderophore ABC transporter substrate-binding protein, and lytic polysaccharide monooxygenase) were shortlisted for epitope prediction. Among predicted epitopes, ten epitopes—GPADGRIAS, TTINHGGAQA, SERTALSVTT, GDGGNGGGEV, GIKEPDLEK, KQADDRIEA, QAIGGDTSN, EPLDEQTASR, AQWEPQSIEA, QPLKFSDFEL—were selected for multi-epitope vaccine construct designing. The screened B- and T-cell epitopes were joined with each other via specific linkers and linked to the cholera toxin B subunit as an adjuvant to enhance vaccine immune protection efficacy. The designed vaccine construct induced cellular and humoral immune responses. Blind docking with immune cell receptors, followed by molecular dynamic simulation results confirms the good binding potency and stability of the vaccine in providing protection against the pathogen.

## 1. Introduction

In the past few decades, species of genus *Enterococcus* emerged as indispensable healthcare-associated bacterial pathogens [1]. The overuse and misuse of antibiotics have undeniably contributed to the increased prevalence of antibiotic-resistant pathogen strains both in the hospital and community milieu [2]. The emergence of resistance in enterococci to vancomycin, penicillin, and also the high-level resistance to aminoglycosides have been reported by previous studies [3,4,5]. Several species of enterococci have been identified to show extensive resistance to clinically used antibiotics. For example, *Enterococcus faecium*, *E. faecalis*, and *E. durans* are notable antibiotic-resistant enterococci species. It has been reported that 99.1% of enterococcus species show resistance to nalidixic acid, 98.6% to kanamycin, and 78.4% to rifampicin. In addition, the genera species are resistant to ciprofloxacin, ampicillin, tetracycline, erythromycin, penicillin G, gentamycin, chloramphenicol, and streptomycin [6]. From a genetic point of view, the resistance genes to commensals and other pathogenic bacteria are transferred via plasmid and transposons [7]. Among all these antibiotic- resistant enterococci, *E. mundtii* shows its involvement in different hospital-related infections [8]. *E. mundtii* was discovered in 1986 as non-motile and yellow-pigmented bacteria extracted from cow teats, soil, and plants. Since then, these species have been isolated from environmental and human sources [9]. According to the research carried out by Kaufold and Ferrieri, two strains of *E. mundtii* from chronic abscesses and sinus mucosa were reported, which was the first case that reported *E. mundtii* as a pathogenic bacteria [10]. The bacteria *E. mundtii* might be a dangerous threat to public health in the future. The mortality rate of the bacteria in an ill patient may exceed 50% [11]. As the resistance determinants of the bacteria are evolving fast and as no vaccine is available against the pathogen, there is an urgent need to identify potential vaccine antigens from the pathogen genome for vaccine development and assist experimentalists in a successful vaccine design [12]. Computational studies tried to assess the human humoral and cellular immune responses [13] and suggested that the identification of proteins with good vaccine properties can increase the probability of discovering a safe vaccine and may provide protection against antibiotic-resistant pathogens [14]. In the present study, to prevent *E. mundtii* infections, a computer-aided multi-epitope-based vaccine is designed [15].

Different computational multi-epitope vaccines have been designed against different bacterial pathogens, such as *Morganella morganii* [16], *Acinetobacter baumannii*, *Pseudomonas aeruginosa* and *Providencia rettgeri*, etc. [17,18]. An ideal multi-epitope vaccine construct consists of a series of overlapping epitopes. The epitopes should have properties such as being antigenic, non-allergic, nontoxic, and good water soluble peptide fragments, which can elicit both a cellular and a humoral immune response against the targeted pathogens [19]. The multi-epitope vaccines have attracted global attention because of the following characteristics: (i) comprises multiple MHC-restricted epitopes that can be recognized by T-cell receptor (TCRs) of multiple clones from various T-cell subsets; (ii) it contains cytotoxic T-cell, helper T-cells, and B-cell epitopes that can induce strong cellular and humoral immune response simultaneously; (iii) consist of multiple epitopes from different targeted organisms; (iv) introduction of adjuvants can enhance the immunogenicity and long-lasting immune responses; and (v) reduce unwanted components that can trigger either pathological immune responses or adverse effects [20].

This study uses a series of immune-informatics approaches applied on the complete proteomic dataset of *E. mundtii* for developing a multi-epitope vaccine [21]. The designed vaccine was then used in different bioinformatics and biophysics approaches to investigate vaccine binding with different host immune receptors and understand its binding mode and interactions. Further, the host immune system was simulated against the vaccine to decipher which type of immunity plays a critical role in clearing the pathogen [13].

## 2. Research Methodology

**Figure 1:**
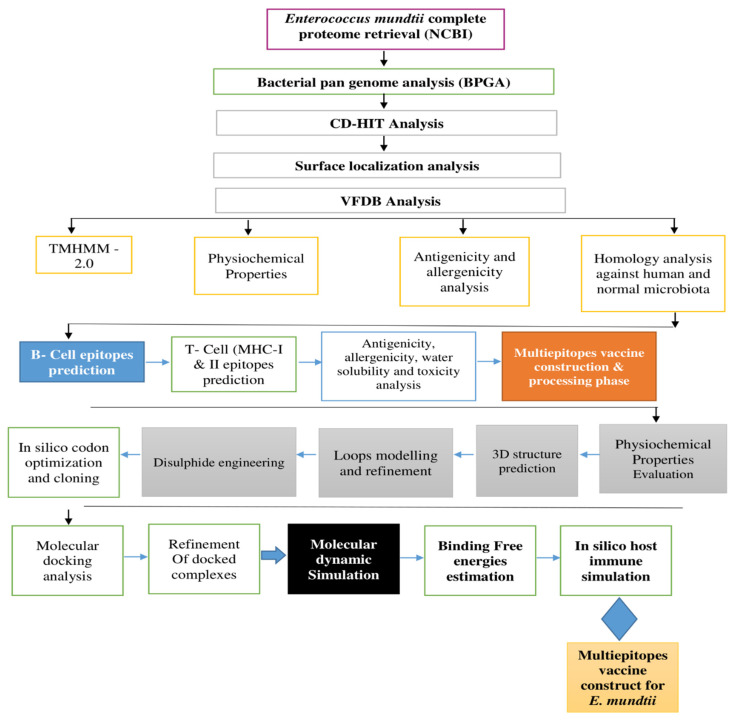
Flow diagram followed for the designing of a multi-epitope vaccine against *E. mundtii*.

The schematic diagram followed for a chimeric vaccine against *E. mundtii* is demonstrated in Figure 1.

### 2.1. E. mundtii Complete Proteome Extraction and Analysis

In the first phase of the study, complete sequenced genomes of *E. mundtii* (two in number at time of the research) were extracted from the NCBI genome database [22]. The extracted genomes were then subjected to a bacterial pan-genome analysis [23,24] using a bacterial pan genome analysis (BPGA) tool to analyze total core proteins [25]. Fast clustering using USEARCH tool of BPGA with a sequence identity cut-off value of 30% was performed, and the resulting core sequences file was next considered for redundant and non-redundant analysis using the CD-HIT web server [26]. Only non-redundant proteins were further subjected to the next steps. Surface localization analysis was performed using the PSORTb 3.0 online tool [27]. In the surface localization check, the outer membrane, extracellular, periplasmic, and cytoplasmic membrane proteins were predicted. All the cytoplasmic membrane proteins were discarded, while outer, extracellular, and periplasmic membrane proteins were subjected to virulent factor database (VFDB) database analysis [28]. The surface localized proteins are exposed to the host immune system and contain antigenic determinants, thus being considered as the best candidate for vaccine designing. Moreover, they play a vital role in the attachment to host cells, infection, and survival of the pathogen [16]. In the VFDB analysis, virulent proteins were predicted with a bit score ≥ 100 and sequence identity ≥ 30%. All the virulent proteins were further subjected to a transmembrane helices analysis using TMHMM-2.0 [29]. In this analysis, the proteins that had more than one transmembrane helix were discarded. In a physicochemical properties analysis, the number of amino acids, molecular weight, and instability index were determined using the online ProtParam Expassy (accessed on 10 January 2022) [30]. To find out the antigenic targets for epitope prioritization phase, VaxiJen 2.0 was utilized, with a threshold value set to 0.4 [31]. Additionally, to avoid autoimmune reactions, an allergenicity analysis of the filtered proteins was performed using Allertop 2.0 [32]. Human and normal microbiota homologous proteins can cause autoimmune reactions. To avoid this, BLASTp check of the filtered vaccine targets against human (tax id:9606) and normal microbiota (*Lactobacillus rhamnosuss* (tax id:47715), *Lactobacillus casei* (tax id:1582), *Lactobacillus johnsonii* (tax id:33959)) was performed [33].

### 2.2. Epitope Prediction and Processing

The immune epitopes database and analysis resource (IEDB) webserver was used for the prediction of B-cells and MHC-II and MHC-II epitopes [34]. For B-cell epitope prediction, the Bepipred linear epitope prediction 2.0 server [35] was used, with a threshold value set to 0.5. In T-cell epitope prediction, both MHC-II and MHC-II epitopes were predicted using the IEDB-recommended 2020.09 method and IEDB-recommended 2.22 method, respectively. In both the MHC epitopes’ prediction phase, a reference set of MHC alleles was used. The predicted epitopes having the least percentile score were considered for further analysis. In the epitope processing, epitopes were analyzed for antigenic probability, allergenicity, water solubility, and toxicity to evaluate epitopes’ potential to provoke proper immune responses, avoid allergic reactions, select water soluble peptides, and prevent toxic responses, respectively [36]. These analyses were conducted using the VaxiJen 2.0 [31], Allertop 2.0 [32], INNOVAGEN [36], and toxin-pred [37], respectively. The vaccine we designed should have wide human population coverage; therefore, the IEDB population coverage analysis tool was used to examine worldwide human population coverage by the predicted epitopes [38].

### 2.3. Multi-Epitope Vaccine Construction and Processing Phase

In the multi-epitope vaccine construction phase, good B-cell and MHC-II and MHC-II binder’s epitopes were linked to each other with GPGPG linkers and joined with cholera toxin B (CTBS). The CTBS was used as an adjuvant to enhance the immune protection efficacy of the designed vaccine [36]. Moreover, physicochemical properties of the designed multi-epitope vaccine construct were evaluated using ProtParam Expassy [30]. Additionally, the designed vaccine model was then subjected to loop modelling, loops refinement, in silico codon optimization, and cloning to check the maximum level of expression [18].

### 2.4. Structure Prediction, Loops Modelling, Refinement, Codon Optimization, and Cloning

The three-dimensional structure of the designed vaccine model was predicted using scratch predictor tools [39]. More loops in the 3D structure of the protein can affect the stability of the protein. However, to improve the structure quality and protein stability, the 3D structure was subjected to a Galaxy server for loop remodeling and structure refinement [40]. The purpose of performing computational cloning was to analyze the expression of vaccine in strains k12 of *Escherichia coli*. First, a designed vaccine sequence was converted into a DNA sequence using the JCAT tool [41]. The value measured was close to 1.0, and the GC value was acceptable at 60. Next, the DNA sequence of the vaccine was cloned in the pET28a (+) vector. Moreover, to improve the structure stability of the predicted structure, several disulfide bonds were incorporated in the designed vaccine construct via the design v2.0 webserver [42].

### 2.5. Molecular Docking Interaction Analysis

The best docked vaccine conformation to host immune cells receptors is mandatory to generate protective immune responses. Herein, docking studies of the vaccine to different immune receptors were performed to analyze the binding interactions of designed vaccine molecules to MHC-I (PDB ID; 1L1Y), MHC-II (PDB ID; 1KG0), and TLR-4 (PDB ID; 4G8A) immune cell receptors. This was accomplished using PatchDock software (Tel Aviv University, Tel Aviv, Israel) [43]. The docked solutions were refined for errors in FireDock [44], and the only lowest global binding energy was selected for a molecular dynamics simulation analysis.

### 2.6. Molecular Dynamic Simulation

To evaluate dynamic behavior of the designed vaccine with the receptors, a molecular dynamics simulation was performed using the same research methodology reported in previous work [45]. This analysis was vital to evaluate and confirm intermolecular stability of the vaccine to the human immune receptors, such as the MHC-I, MHC-II, and TLR-4 receptor. The parameter file for both vaccine and receptors was generated using the AMBER20 [45] antechamber program. Force field, ff14Sb [46] was used in processing the molecules and making them ready for a simulation production run of 500 ns. The simulation protocol was divided into energy minimization, heating, equilibration, and production run. CPPTRAJ was [36] used to analyze simulation trajectories.

### 2.7. Free Binding Energies Calculation

Next, molecular mechanics energies combined with the Poisson–Boltzmann or generalized Born and surface area continuum solvation (MM-PB/GBSA) approach was used to estimate binding free energies of docked vaccine–immune receptors [47]. The binding energies calculation throughout the simulation system was performed using 1000 frames picked at a regular interval from simulation trajectories.

### 2.8. Host Immune Simulation

In order to decipher host immune system responses against a vaccine antigen, the C-ImmSim simulation server (http://150.146.2.1/C-IMMSIM/index.php, accessed on 10 January 2022) was used. In total, three injections of the vaccine were administered at 4 weeks apart. The rest of the parameters were used as the default (random seed = 12345 and vaccine without containing LPS).

## 3. Results

### 3.1. Subtractive Proteomics Analysis

**Figure 2:**
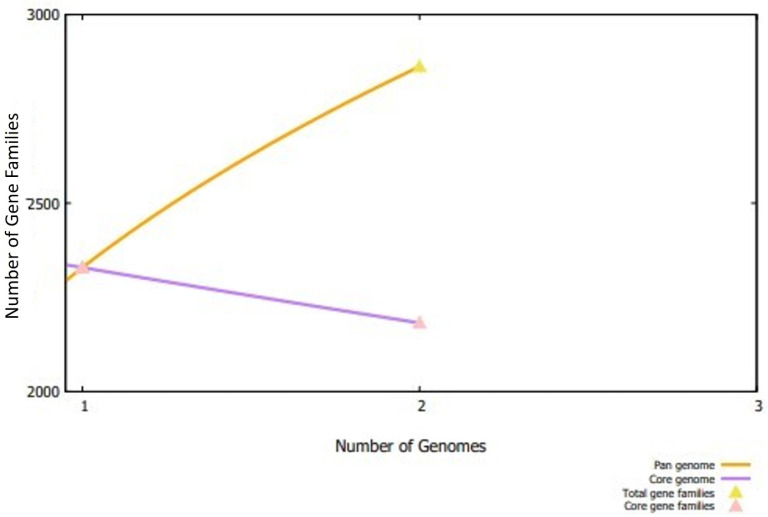
Core pan plot of two *E. mundtii* strains. The figure shows the number of gene families in each strain genome.

In the first phase of the current study, two complete genomes of *E. mundtii* were retrieved from the NCBI genome database and further subjected to BPGA, and it was found that *E. mundtii* strains comprise 4326 core proteins, with an average of 2181 core proteins in each strain [25]. Meanwhile, the average number of absent, accessory, and unique proteins were noted as 340, 0, and 340, respectively. The number of proteins of each *E. mundtii* is presented graphically in Figure 2.

### 3.2. CD-HIT, Surface Localization, VFDB, Antigenicity, Allergenicity, Homology, Transmembrane Helices, and Physiochemical Properties Analysis

**Figure 3:**
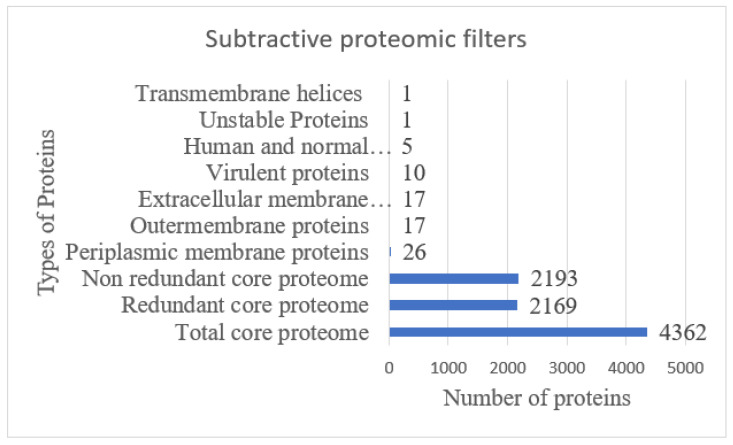
Number of proteins achieved at each step of subtractive proteomics, starting from the core genome.

A CD-HIT analysis revealed that out of 4362 total core proteins, 2169 were found as redundant proteins and 2193 were non redundant [48]. The non-redundant proteins were subjected to a localization analysis. Out of 2193 non redundant proteins, 17 were outer membrane (OMPs), 17 were extracellular membrane, and 26 were periplasmic membrane proteins [49], as shown in Figure 3. Out of 60 subcellular localized proteins, 10 were predicted as virulent, non-allergic, and a probable antigen with a score > 0.4. Further, five proteins were homologues to human and intestinal microbiota, one was unstable, and one protein had more than one transmembrane helix, and hence was discarded from the study and mentioned in Figure 3. The core proteins as shared by both strains can be good targets for a broad-spectrum vaccine design [50]. The surface localized proteins are attractive vaccine targets because of their direct interaction with the host immune system and contain antigenic determinants [51]. The virulent proteins, on the other hand, stimulate defense pathways of the host. The host non-homologous proteins provide the opportunity to avoid autoimmune reactions [52]. Similarly, a lesser number of transmembrane helices ensures only those proteins should be selected, which can be easily cloned and expressed [18].

### 3.3. B-Cell Derived T-Cell Epitope Prediction

To generate humoral and cellular immunity, both B- and T-cell (MHC-I and II) epitopes were predicted, as they are involved in antigen processing and presentation [53]. One extracellular (lytic polysaccharide monooxygenase) and two periplasmic proteins (siderophore ABC transporter substrate-binding protein and lytic polysaccharide monooxygenase) were shortlisted for the epitope prediction phase. Form (lytic polysaccharide monooxygenase) and seven epitopes from siderophore ABC transporter substrate-binding protein and lytic polysaccharide monooxygenase, and four and two epitopes were predicted, respectively, as tabulated in Table 1. Moreover, in T-cell epitope prediction steps, both MHC-I and MHC-II epitopes were predicted, and only those epitopes were considered best for a multiple-epitope vaccine, having the least percentile score as shown in Supplementary Materials Table S1.

### 3.4. Multi-Epitope Vaccine Construction and Processing Phase

In the multi-epitope vaccine construction phase, a total of 10 epitopes (GPADGRIAS, TTINHGGAQA, SERTALSVTT, GDGGNGGGEV, GIKEPDLEK, KQADDRIEA, QAIGGDTSN, EPLDEQTASR, AQWEPQSIEA, and QPLKFSDFEL) were screened as probable antigenic, non-allergic, non-toxic, and good water-soluble predicted epitopes were joined with each other via “GPGPG” linkers to make a multi-epitope vaccine construct. The designed vaccine construct was additionally linked with CTBS adjuvant to make the construct more potent to stimulate strong immunological responses [16]. The above mentioned linkers are rigid and avoid folding of the epitopes [54].

### 3.5. Physiochemical Properties, 3D Structure, Loops Modeling and Refinement, and Disulphide Engineering

**Figure 4:**
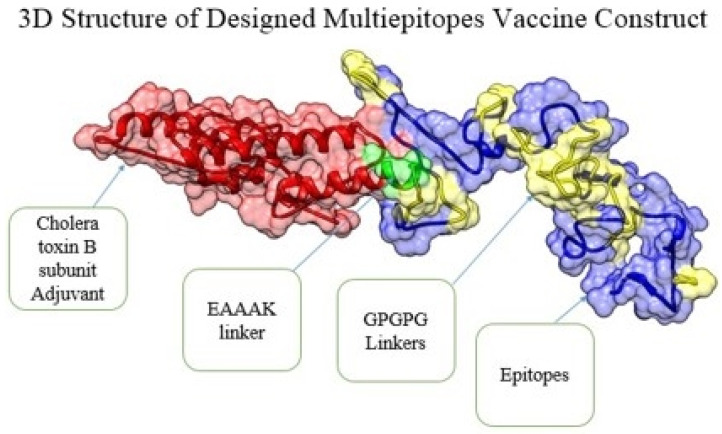
Tertiary structure of the designed vaccine construct. Epitopes are colored blue, adjuvant is in red, and EAAAK linkers and GPGPG linkers are in green and yellow, respectively.

Before designing the three-dimensional structure of the multi-epitope vaccine construct, physiochemical properties were checked. The vaccine consists of 275 amino acids, having molecular weight, theoretical PI instability index, aliphatic index, and GRAVY of 28057.26 da, 5.3, 27.83, 63.64, and −0.494, respectively. The 3D structure of the designed multi-epitope vaccine model was required for the next phases [55]. Herein, a 3D structure prediction was used to see the theoretical vaccine structure and understand its binding, with different immune receptors in afterward steps. The model vaccine is mentioned in Figure 4. Furthermore, for structure stability, all the loop regions—Cys30-Gln37, Ser51-62ile, Phe63-73val, Leu98-Leu106, Lys129-Gly148, Thr149-Leu169, Ser170-Asn183, Val188-Gly193, Gly194-Arg213, Ile214-Gly226, Asn230 Glu236, Pro237-Glu254, and Ile258-Lys259—were modeled [56]. Moreover, the loops’ modeled structure was submitted to refinement, and the best model was selected among the models tabulated in Table 2. Model 1 was selected as the best model due to a good root mean square deviation (RMSD) value, ramachandran favored regions, and improved galaxy energy value. Low RMSD indicates the high structure quality, residues compactness, and less deviation from the actual 3D structure.

**Figure 5:**
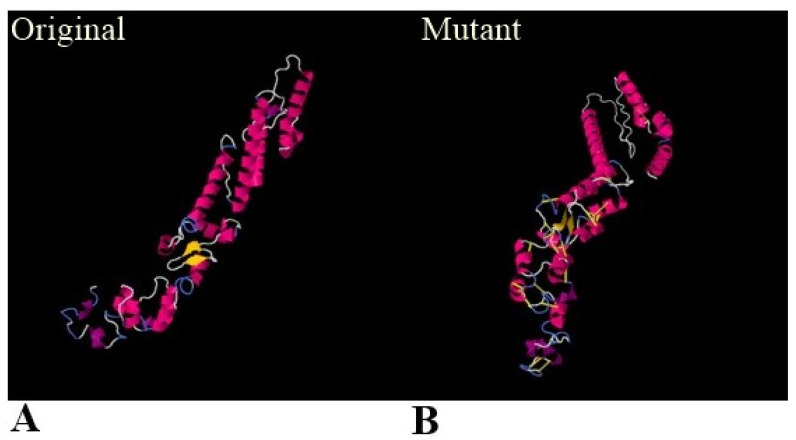
Disulfide engineering of the designed vaccine structure. (**A**) Wild vaccine structure before disulfide engineering. (**B**) Disulfide engineered structure with disulfide bonds shown in yellow sticks.

Moreover, disulfide bonds were created via the disulfide engineering approach to optimize the stability and molecular interactions of the vaccine construct [36]. Twenty-two pairs of residues were predicted to replace with cysteine amino acids, as mentioned in Table 3, and represented by a yellow-colored stick in the mutated structure given in Figure 5.

### 3.6. In Codon Optimization Cloning and Population Coverage Analysis

**Figure 6:**
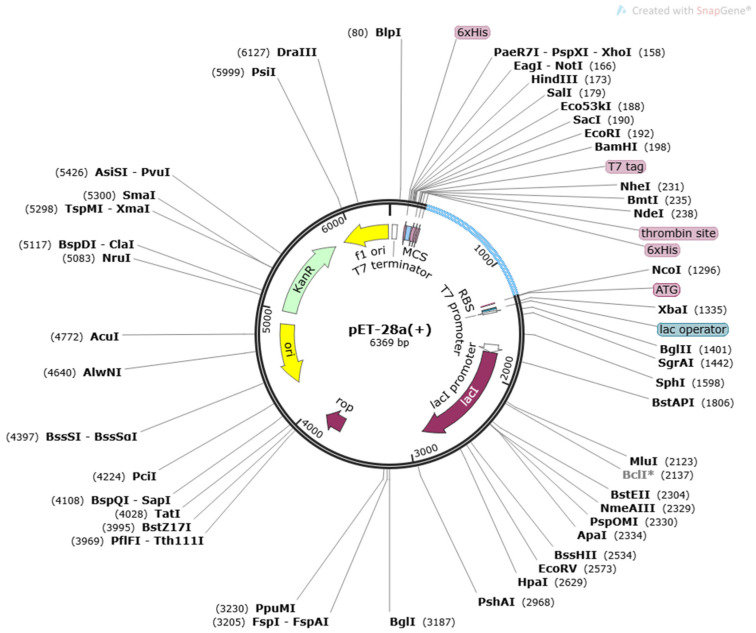
Cloning of designed vaccine into a pET28a (+) vector. Inserted DNA segment is shown in a light blue color, while the plasmid is in black.

Codon optimization is a genetic procedure for the optimization of a specific sequence as per translation machinery of the host to obtain maximum expression in the host. Codon optimization of the vaccine was measured through “codon adaptation index value (CAI)”, which is 0.9741, and its GC content is 56.1212%. The CAI and GC content values represent the effective codon usage of the model vaccine construct sequence into the *E. coli* K12 strain. In the last, the engineered optimized vaccine model was inserted into vector pET-28a (+), as shown in a light blue color (Figure 6). The SnapGene tool [54] was used for plasmid designing and is depicted in Figure 6.

**Figure 7:**
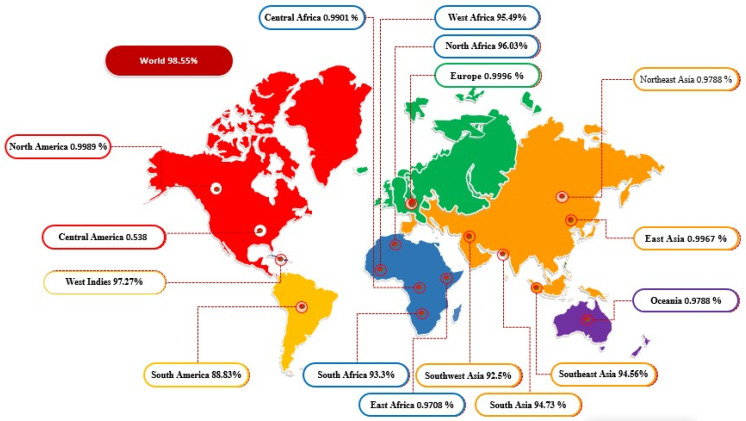
World- and country-based population coverage of the designed vaccine construct. The figure provides a general idea of how much the designed vaccine epitopes cover the world populations.

In the population coverage analysis, combined CTL and HTL epitopes showed 98.55% worldwide human population coverage. Country-wise population coverage of the predicted vaccine construct is shown in Figure 7. The designed vaccine epitopes are reflected to provide maximum protection to the population of North Africa, South Africa, Southeast Asia, Southwest Asia, South Asia, South Africa, South America, and the West Indies.

### 3.7. Molecular Docking and Refinement Studies

**Figure 8:**
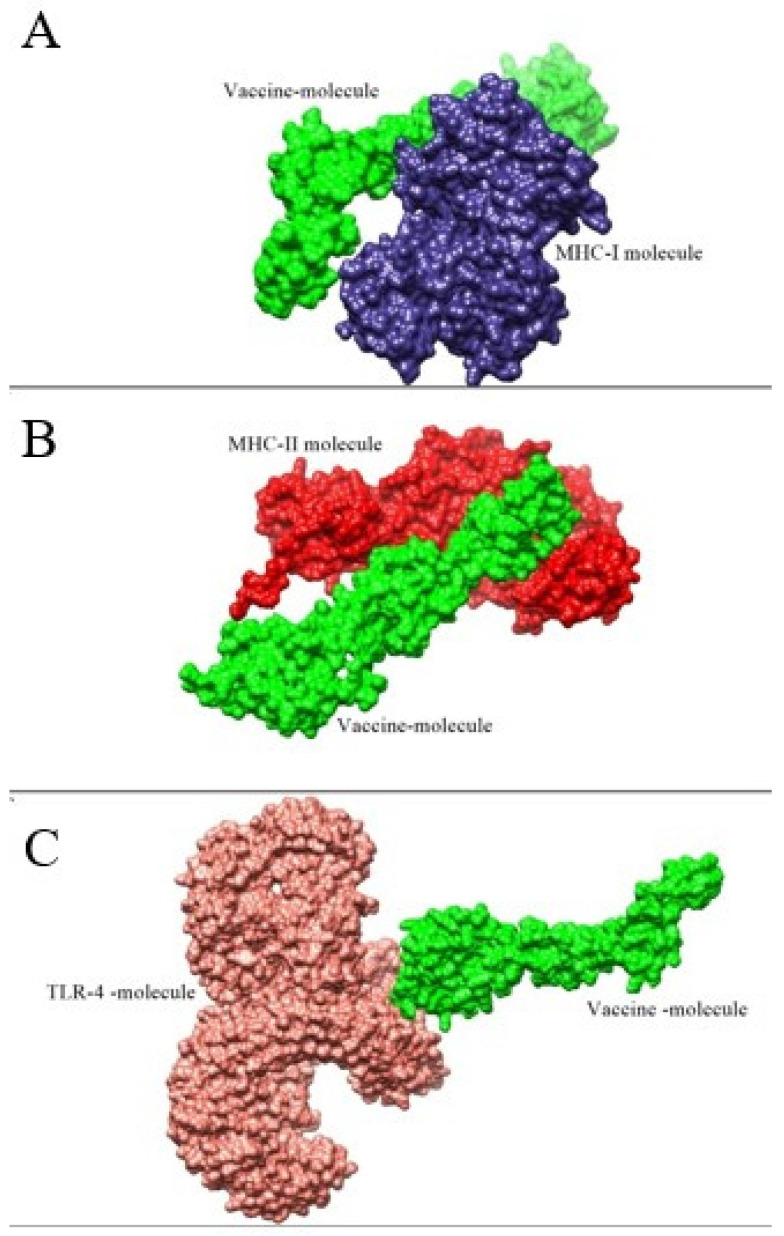
Molecular docking analysis of the vaccine with immune receptors. (**A**) The green color represents the vaccine molecule, while the blue color represents MHC-I molecule. (**B**) The green color represents MHC-II molecules, while the red color represents the vaccine molecule. (**C**) The green represents the vaccine molecule, and the peach puff color shows human TLR-4 receptors.

Docking studies were performed for the evaluation of binding affinity of the vaccine to immune cell receptors, MHC-I, MHC-II, and TLR-4 [57]. In each case of docking, 20 complexes were found, as mentioned in Supplementary Materials Tables S2–S4. Furthermore, for refinement, the top 10 docked complexes were selected, as given in Supplementary Materials Tables S5–S7. In the case of vaccine and MHC-I molecule, vaccine-MHC-II, and vaccine-TLR-4, the best solution number 10, 9, and 6, respectively, were considered for a molecular dynamic simulation based on least global energy (G.E), attractive VdW (A.VdW), repulsive (VdW) Atomic center energy (ACE), and hydrogen bonding (HB). The intermolecular docked conformation of vaccine and receptors is shown in Figure 8.

### 3.8. Molecular Dynamic Simulation, Hydrogen Bonding, and Free Binding Energies Calculation

**Figure 9:**
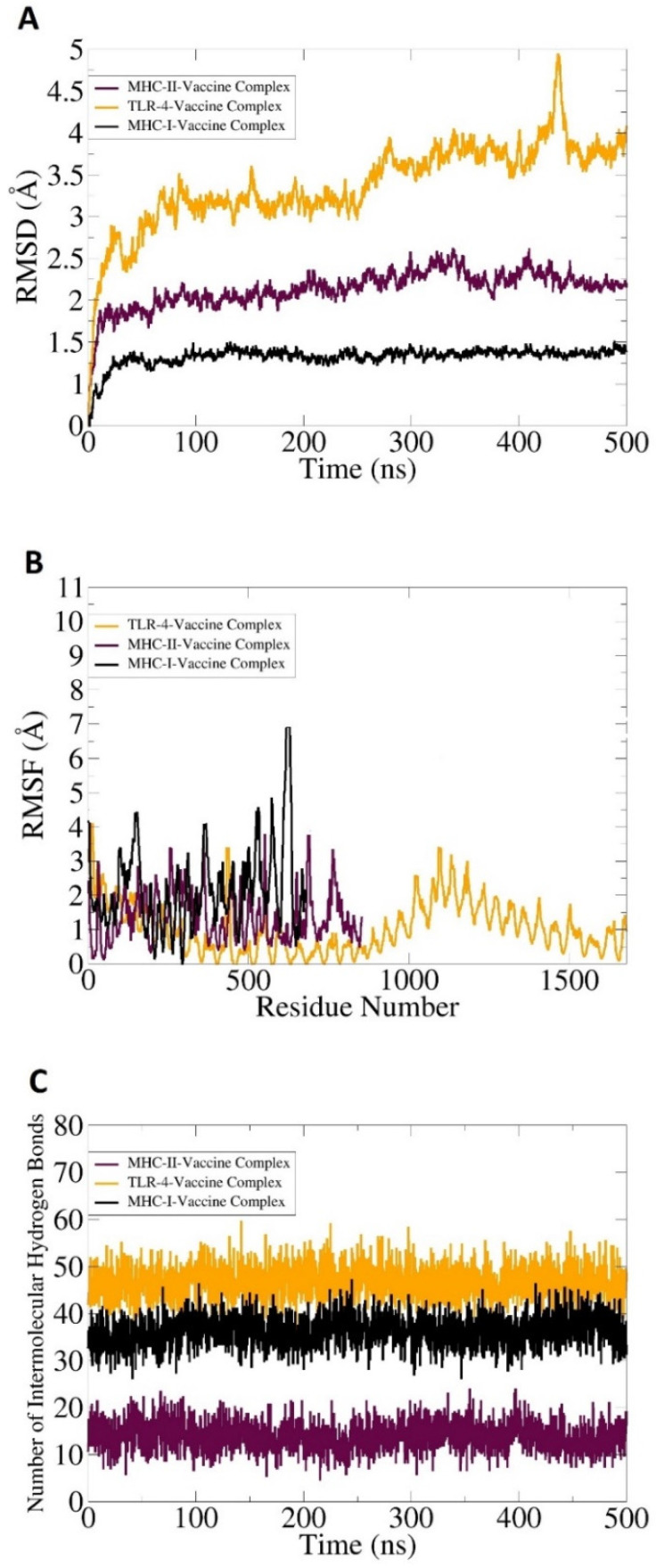
Molecular dynamics simulation analysis of a vaccine molecule docked with different immune receptors. (**A**) Carbon alpha based RMSD, (**B**) carbon alpha based RMSF, and (**C**) number of intermolecular hydrogen bonds formed between vaccine and immune receptors in each frame of simulation trajectories.

A molecular dynamics simulation analysis is critically important to evaluate the stability of a vaccine and immune cell receptors’ docked complex. The AMBER 20 (University of California, San Francisco, USA) [58] software was used to examine the dynamic behavior of docked molecules. The software examined the direction and extent of intrinsic motions of the docked complexes in terms of RMSD, root mean square fluctuation (RMSF), and number of hydrogen bonding. It was crucial to evaluate that the vaccine antigens are well exposed to host immune cells, as it is easily recognizable by the cells of the immune system to generate proper immune responses. No extreme changes were observed throughout the simulation, as represented in Figure 9A–C. In the simulation, RMSD and RMSF were performed based on carbon alpha atoms. Findings of the RMSD and RMSF revealed stable plots. As compared to TLR-4, both MHC-I and II docked complexes showed good binding stability. The mean vaccine-MHC-I and MHC-II complexes RMSD value was ~1 and 1.5 angstroms, correspondingly, while the mean RMSD of the TLR-4 vaccine complex was reported as 5.8 angstroms. Some of the deviations throughout the simulation time were due to the larger size of the complexes and contained several flexible loops. This was evident in the RMSF analysis.

Hydrogen bonds were mainly formed between the electronegative charge particles. Hydrogen bonds are non-covalent forces and are formed between electronegative donors and acceptors [59]. A visual molecular dynamic (VMD) plugin was utilized for counting and the identification of hydrogen bonding between a vaccine molecule and receptors’ molecules formed in simulation, as shown in Figure 9C. The threshold value distance value was 3 Å. In each case, higher and strong intermolecular hydrogen bonds were formed between vaccine-MHC-I (>40), MHC-II, (>30), and TLR-4 (>50), as mentioned in Figure 9C.

Additionally, binding free energies were calculated as a post-simulation process using MM-PB/GBSA. This analysis was conducted to estimate binding free energies of the docked complexes to confirm the binding efficacy of the designed vaccine to immune cell receptors. The different free binding energies of the vaccine-MHC-I molecule, vaccine-MHC-II, and vaccine-TLR-4 molecule, as calculated in MM-GBSA and MM-PBSA [60] methods, are tabulated in Table 4, respectively. The net value predicted in MM-GBSA was −456.67 kcal/mol, −387.17 kcal/mol, and −515.99 kcal/mol for the TLR-4-vaccine complex, MHC-I-vaccine complex, and MHC-II-vaccine complex, respectively. The net MM-PBSA energy value was 469.56 kcal/mol for the TLR-4-vaccine complex, −372.7 kcal/mol for the MHC-I-vaccine complex, and −494.11 for the MHC-II-vaccine complex, as tabulated in the following Table 4.

### 3.9. In Silico Immune Simulation

**Figure 10:**
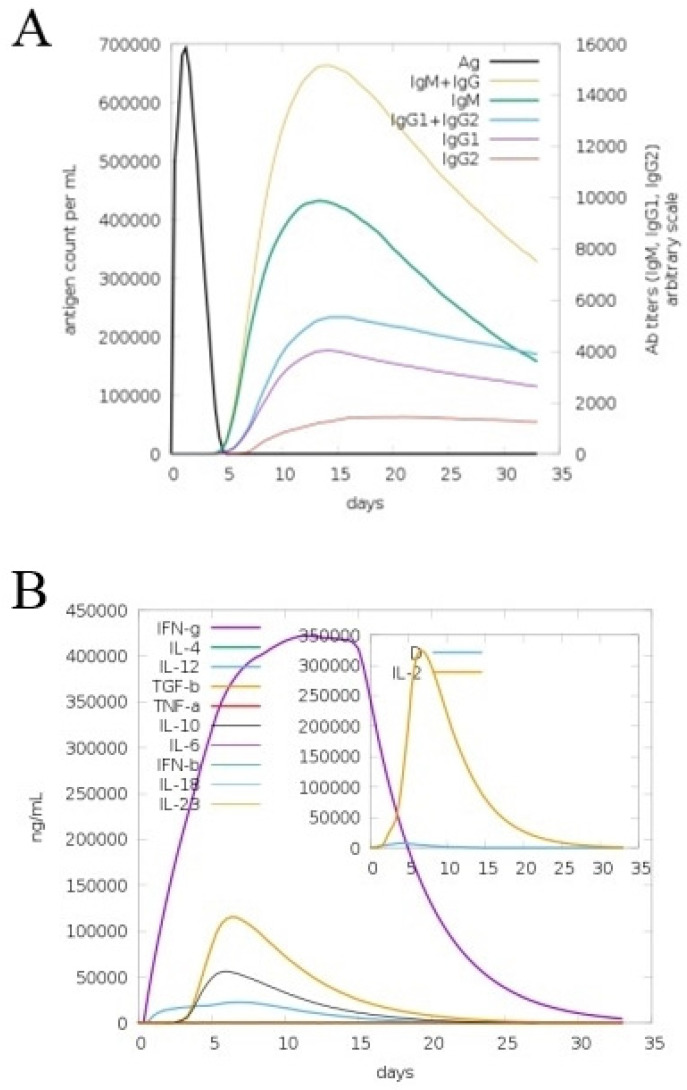
Host immune system stimulation in response to the designed vaccine. Different antibody titers produced to the vaccine antigen (**A**) cytokines and interleukins concentration against the vaccine antigen (**B**).

The C-Immsim server was used to study the human immune system dynamics in response to the vaccine construct. Significant antibody titers were produced against the antigen and shown in Figure 10A. The combined lgM and lgG showed the highest peak reaching a titer of 650,000 antigenic count/mL, followed by an individual lgM with an antibody titer of 400,000 antigenic count/mL. Additionally, the response of lgG1 + lgG2 was also high. Among the cytokines and interleukins, interferon-gamma (IFN-g) showed a robust response, with the scale reaching > 400,000 ng/mL (Figure 10B). The cellular immune response was also excellent, including the formation of memory cells for the pathogen recognition on re-encounter.

## 4. Discussion

*E. mundtii* infections might be a significant threat to public health in the future, and attention on this opportunistic enterococcus bacterial pathogen is required due to its growing antibiotic resistance potential and virulence. Therefore, therapeutic and prophylactic efforts are required to manage infection caused by this bacterial pathogen [8]. In this study, a novel multi-epitope-based vaccine against above said bacterial pathogen is designed using core genome of the pathogen [61]. The BPGA pipeline was used to extract core sequences from a complete proteome and subjected to a CD-HIT analysis to remove redundant proteins. Redundant sequences are not required for vaccine designing due to their double representation in the genome [25]. Surface localized protein sequences are mainly involved in the pathogenesis and are regarded as good vaccine targets [62]. Next, surface localized proteins sequences were found following the same methodology of [36]. Furthermore, virulent proteins can stimulate and generate a proper immune response, so virulent proteins were predicted using a VFDB analysis. For an easy experimental analysis and cloning and expression analysis, all the proteins were discarded, having more than one transmembrane helix [18]. Moreover, an antigenicity, allergenicity, and homology analysis were conducted following the same methodology of Khan et al. 2020 [46]. A vaccine target similar to human and intestinal beneficial flora of humans can cause severe autoimmune reactions, while vaccine targets having an allergic nature may cause unwanted allergic reactions [48]. Antigenic proteins have the capability of stimulating host immune responses, especially generating cellular immunity. Therefore, in the current study, we considered those proteins for epitope predication, which were probable antigenic, non-allergic, and non-homologues to human and normal microbiota species. In the epitope prediction phase, both B- and T-cell epitopes were predicted in order to generate both humoral and cellular types of immunity [63]. Only antigenic, non-allergic, nontoxic, and good water soluble top 10 epitopes were utilized to design a multi-epitope-based vaccine. Multi-epitope-based vaccines consist of several overlapped epitopes, which help in the prevention of many infectious diseases [54]. A problem that may occur in the designing of a multi-epitope vaccine includes the appropriate selection of epitopes. Therefore, for the appropriate selection of epitopes, the above said analysis of allergenicity, toxicity, antigenicity, and water solubility were assessed for shortlisted epitopes. Subsequently, the population coverage of the designed vaccine was analyzed and showed a 98.55% coverage in the combined approach (MHC-I and MHC-II). The filtered epitopes further joined to each other and linked via another EAAAK linker with a cholera toxin B subunit adjuvant. The adjuvant was used to boost up the efficacy of the designed vaccine construct, as in a previous study [64] that also designed a multi-epitope vaccine construct against *Mycobacteroides abscessus* using the same methodology [65]. Binding of vaccine construct molecules with host immune receptors is necessary to activate a cellular and humoral immune response against the target pathogen [66]. A docking study approach was used for the prediction of binding efficacy of a vaccine construct with immune cell receptors [67]. The docked complexes may lose the stability vs. time and are not able to activate a proper immune response. For further validation of a dynamic behavior of docked molecules, molecular dynamics simulations were applied in which a RMSD, RMSF, and hydrogen binding analysis were conducted [68]. Previously, [16] also conducted a molecular dynamics simulation analysis in order to check the dynamic behavior of the docked complexes. Free binding energies also suggested strong binding energies. This ensures that the vaccine molecule has good binding efficacy with immune cell receptors; hence, the designed vaccine has the capability of generating immune responses [36].

## 5. Concluding Remarks

*E. mundtii* is an emerging multi-drug-resistant enterococcus species and has the ability to cause serious infections in the future [69]. Formulation of a vaccine that could prevent humans from infections of the *E. mundtii* would be ideal. Due to the complex biology and genetic variation of the above said pathogen, a daunting nature of pasture vaccinology role and vaccine formulation against this antibiotic-resistant pathogen is challenging. Hence, we formulate a computer-aided multi-epitope vaccine against *E. mundtii* based on probable antigenic epitopes retrieved from three proteins: lytic polysaccharide monooxygenase, siderophore ABC transporter substrate-binding protein, and lytic polysaccharide monooxygenase prioritized from the pathogen’s core antigenic proteins to develop a broad-spectrum effective vaccine candidate. All the predicted epitopes were screened for antigenicity, allergenicity, water solubility, and toxicity, and probable antigenic, non-allergic, non-toxic, and good water-soluble epitopes were used in vaccine designing. The designed vaccine molecule has been demonstrated to evoke both major antibody-dependent and cellular-dependent immune responses. Particularly, to avoid unwanted allergic immune reactions, the designed vaccine epitopes are non-homologous to the human host and normal microbiota species. The designed vaccine covers all good properties of a vaccine and is showing good binding affinity and stability to MHC-I, MHC-II, and TLR-4 immune cell receptors [70].

## Figures and Tables

**Table 1:** Predicted B-cell epitopes from each shortlisted protein.

Proteins Accession Number/Name	Predicted B-Cell Peptides
>core/490/2/Org2_Gene1203 (lytic polysaccharide monooxygenase)	GSLDRNVNHNAALAKYGPVIYEPQSLEALKGFPQAGPADGRIASANGAVGNNFNLDRQTSTMWTKQDLNTG
	ADWNPNDQLDRSDFELLTTINHGGAQASTN
	VDDTAMAFYQVIDVNLKGDSAIPVAPTAPRNVRTTNVTSS
	LLGNTASPDFSDQNLTAE
	QTGLVSERTALSVTTLSETTEEKPTAPSHL
	DRDENGGGDENGGDGGNGGGEVVTGRQWTVGSFFSPVS
	ITWQSHLNYGDTNWAPGIAHSL
>core/1058/1/Org1_Gene1225 (siderophore ABC transporter substrate-binding protein)	NEAQTRETTASSTIATD
	NLPAYLEKYQEVESAGGIKEPDLEKINEM
	KQADDRIEASTHGQSVSYEYVL
	TQAIGGDTSNDN
>core/1868/1/Org1_Gene902(lytic polysaccharide monooxygenase)	GNLNQNVGRAQWEPQSIEAPKNTFIDGKIASAGVSGFEPLDEQTASRWHKSVINSGA
	PGWNQNQPLKFSDFELITKIDDKATIPP

**Table 2:** Different refined models and their structure information. Root mean square deviation (RMSD).

Structure Information						
Model	RMSD	MolProbity	Clash Score	Poor Rotamers	Rama Favored	GALAXY Energy
Initial	0	2.99	39.1	3	89.7	13,957.59
MODEL 1	2.01	1.199	0.5	0	90.5	−4775.14
MODEL 2	2.156	1.483	1.9	0.5	90.5	−4771.87
MODEL 3	2.724	1.214	0.7	0.5	91.9	−4765.4
MODEL 4	2.272	1.352	1.2	0	90.8	−4764.62
MODEL 5	2.675	1.329	1.2	0	91.6	−4754.89
MODEL 6	2.227	1.25	0.7	0	90.8	−4751.34
MODEL 7	2.208	1.214	0.7	0	91.9	−4750.35
MODEL 8	2.093	1.315	0.9	0	90.5	−4748.02
MODEL 9	2.478	1.341	1.2	0	91.2	−4747.58
MODEL 10	2.095	1.326	0.9	0.5	90.1	−4746.33

**Table 3:** Predicted unstable pair residues, with high unfavorable energy selected for disulfide engineering.

A.A Residues Pairs	Chi3 Value	Energy	Sum B-Factors
GLU32-ASN35	126.99	4.04	0
MET 89-PRO114	−116	5.52	0
GLU 100-GLY161	103.67	2.53	0
LYS 129-GLY134	99.26	3.2	0
ALA 137-ARG140	79.22	4.58	0
ASN152-GLY181	114.66	2.76	0
GLY155-ASP180	74.31	4.37	0
THR 173-GLY 217	121.34	3.34	0
GLY 174-GLY193	−57.19	4.85	0
GLY179-ASN183	100.56	2.89	0
GLY189-GLY217	−100	4.12	0
PRO190-GLY219	−94.95	4	0
LYS196-ASP199	125.12	5.84	0
LYS202-LYS208	85.73	3.3	0
PRO204-GLY231	−85.8	4.06	0
GLY205-LYS208	74.84	2.52	0
ALA210-GLU236	93.44	0.73	0
GLN222-GLY226	68.24	6.37	0
PRO237-THR242	94.65	2.39	0
SER257-GLY263	107.81	5.71	0
GLY261-GLN266	107.38	0.93	0
PRO262-PHE270	113.73	2.28	0

**Table 4:** Binding free energies of docked complexes calculated via MM-PBSA and MM-GBSA analysis. All energy values are provided in kcal/mol.

Energy Parameter	TLR-4-Vaccine Complex	MHC-I-Vaccine Complex	MHC-II-Vaccine Complex
**MM-GBSA**			
van der Waals	−397.98	−306.66	−407.08
electrostatic	−115.66	−112.37	−128.39
polar	81.97	62.09	39.14
non-polar	−25.00	−30.23	−19.66
gas phase	−513.64	−419.03	−535.47
solvation	56.97	31.86	19.48
net	−456.67	−387.17	−515.99
**MM-PBSA**			
van der Waals	−397.98	−306.66	−407.08
electrostatic	−115.66	−112.37	−128.39
polar	73.12	71.00	58.51
non-polar	−29.04	−24.67	−17.15
gas phase	−513.64	−419.03	−535.47
solvation	44.08	46.33	41.36
net	−469.56	−372.7	−494.11

## Data Availability

The data presented in this study are available within the article.

## Supplementary Materials

The following are available online at https://www.mdpi.com/article/10.3390/ijerph19063729/s1, Figure S1. A. Different B-cell counts in response to the designed vaccine model. B. Different types of cellular immune response toward designed vaccine especially in the form of T-cell responses i.e. cytotoxic killer T-cells (Tc), natural killer cells (NK), dendritic cells (DC), macrophages (Mφ), and epithelial cells; Table S1: Predicted B-cell derived T- cells (MHC-I and MHC-II) Epitopes with their least percentile score; Table S2. Docking results of MHC-I Vaccine complex; Table S3. Docking results of MHC-II vaccine complex; Table S4. Docking results of TLR4- vaccine complex; Table S5. Top 10 refine docked complex of MHC-I vaccine generated by fire dock server; Table S6. Top 10 refine docked complex of MHC-II vaccine generated by fire dock server; Table S7. Top 10 refine docked complex of TLR-4vaccine generated by fire dock server.
