# Supplementary material for: Designing of a Recombinant Multi-Epitopes Based Vaccine against Enterococcus mundtii Using Bioinformatics and Immunoinformatics Approaches
Source: Int J Environ Res Public Health. 2022 Mar 21;19(6):3729. doi: 10.3390/ijerph19063729 (PMC8949936; doi:10.3390/ijerph19063729)
Supplement: Supplementary file 1 [file ijerph-19-03729-s001.zip › ijerph-1605769-supplementary.pdf]

Designing of a Recombinant Multi-epitopes based Vaccine against *Enterococcus mundtii* Using Bioinformatics and Immunoinformatics Approaches

Table S1: Predicted B-cell derived T- cells (MHC-I and MHC-II) Epitopes with their least percentile score

MHC-I	Percentile Score	MHC-II	Percentile Score
NVNHNAALAK	0.93	GSLDRNVNHNAALAK	1.7
GSLDRNVNH	1.3		
GPVIYEPQSL	0.35	KYGPVIYEPQSLEA	13
GPADGRIAS	0.5	GFPQAGPADGRIASA	7.7
GFPQAGPAD	44		
AVGNNFNLDR	1.4	NGAVGNNFNLDRQT	31
STMWTKQDL	1.4	QTSTMWTKQDLNTG	45
TTINHGGAQA	0.73	TTINHGGAQASTN	0.42
ADWNPNDQL	0.55	ADWNPNDQLDRSDFE	41
DQLDRSDFE	21		
NLKGDSAIPV	1.2	DVNLKGDSAIPVAP	1.8
APRNVRTTNV	0.04	TAPRNVRTTNVTSS	16
DTAMAFYQV	0.02	VDDTAMAFYQVIDV	14
SPDFSDQNL	0.02	NTASPDFSDQNLTAE	23
GLVSERTAL	0.48	QTGLVSERTALSVTT	14
SERTALSVTT	3.1	LSETTEEKPTAPSHL	43
EEKPTAPSHL	0.25		
LSETTEEKP	8.7		
GGGEVVTGR	1.7	GDGGNGGGEVVTGR	1.9
GDGGNGGGEV	38		
RQWTVGSFF	0.03	GRQWTVGSFFSPVS	2.2
DENGGGDEN	12	DRDENGGGDENGGD	71
WAPGIAHSL	0.43	YGDTNWAPGIAHSL	7.2
YGDTNWAPGI	14	ITWQSHLNYGDTNWA	21
ITWQSHLNY	0.14		
HLNYGDTNWA	1.3		
RETTASSTI	0.08	TRETTASSTIATD	4.5

RETTASSTI	0.08	NEAQTRETTASSTIA	15
AYLEKYQEV	0.07	NLPAYLEKYQEVE	8.8
GIKEPDLEK	0.017	GGIKEPDLEKINE	52
STHGQSVSY	0.02	ASTHGQSVSYEYVL	12
QSVSYEYVL	1.7	KQADDRIEASTHGQ	39
KQADDRIEA	0.52		
RIEASTHGQ	12		
QAIGGDTSN	9.3	QAIGGDTSNDN	11
FIDGKIASA	0.07	FIDGKIASAGVSGF	1.1
KIASAGVSGF	0.022		
EQTASRWHK	1	EPLDEQTASRWHKSV	52
EPLDEQTASR	1.6		
AQWEPQSIEA	0.2	GRAQWEPQSIEAPKN	30
QPLKFSDFEL	0.51	NQPLKFSDFELIT	0.17
ELITKIDDK	1.6	ELITKIDDKATIPP	9.17
GWNQNQPLKF	0.3	PGWNQNQPLKFSDF	5.8
NQPLKFSDF	0.72		

Table S2. Docking results of MHC-I Vaccine complex

Solution No	Score	Area	ACE	Transformation
1	17542	2822.3	353.39	-2.91 0.84 -0.20 25.91 25.83 66.99
2	17426	2820.9	187.90	-0.02 -0.53 2.25 40.00 38.45 35.95
3	17202	2315.9	322.02	-0.54 -0.73 1.56 25.96 34.45 21.20
4	16640	2710.6	186.05	2.97 0.51 0.15 30.06 28.62 68.00
5	16634	2375.1	223.24	0.36 0.60 -0.79 57.91 47.81 45.70
6	16458	2266.7	433.43	1.67 0.61 0.89 26.30 26.71 104.85
7	16160	2576.2	251.95	3.09 0.50 0.00 32.66 24.92 70.30
8	15900	2696.4	237.05	0.56 -0.63 1.03 15.18 48.52 41.92
9	15610	2827	230.43	0.59 0.82 -0.85 54.54 49.41 48.07
10	15550	2540.8	288.59	2.40 0.31 -0.82 44.69 8.68 45.45
11	15504	2271.3	485.19	-0.31 -0.44 2.26 38.39 48.97 33.32
12	15424	2540.9	350.58	-0.92 -0.47 -2.98 62.35 33.62 0.99
13	15408	1970.2	252.17	0.48 0.64 -0.82 54.91 49.02 45.30
14	15398	1780.5	79.28	2.40 0.40 0.07 28.93 3.77 57.80
15	15198	2503.4	247.46	1.90 0.25 -2.60 75.49 36.32 36.78
16	15190	2197.7	407.49	-1.34 -0.11 -0.87 53.81 23.47 40.04

17	15124	3263.8	14.45	-0.55 -0.82 1.83 27.71 28.90 19.25
18	15120	2249.6	394.07	2.95 -0.13 -2.65 34.96 5.08 70.43
19	15116	2345.3	247.85	-0.66 -0.88 -2.97 76.39 29.03 9.55
20	15002	2067.4	224.43	0.91 0.16 -1.84 46.36 24.47 47.47

Table S3. Docking results of MHC-II vaccine complex

Solution No	Score	Area	ACE	Transformation
1	19482	2752.3	484.01	-2.86 1.13 -2.96 116.95 105.96 39.26
2	19054	2835.7	397.39	-2.35 1.18 2.86 117.50 111.20 33.06
3	18022	2396.4	57.51	-0.22 -1.25 -2.89 93.50 91.52 17.55
4	17528	2504.4	281.14	2.82 -0.32 3.07 81.25 64.30 35.85
5	17448	3174.4	4.59	-0.21 -1.08 -2.89 96.83 93.63 18.50
6	17156	2622.3	416.37	0.89 0.77 2.89 113.26 59.03 15.39
7	17004	2722.2	53.96	0.98 -0.99 -1.01 136.23 40.84 3.21
8	17004	2527.1	475.51	-2.49 -1.02 1.26 133.75 92.97 18.30
9	16996	2359.5	143.44	2.54 1.04 1.22 119.23 18.43 28.60
10	16976	2734.9	111.71	-2.82 -1.28 0.91 116.79 111.34 28.76
11	16938	2017.3	288.63	-2.68 -0.22 -2.64 67.59 81.85 23.32
12	16900	2437.4	460.95	2.26 0.12 -1.80 102.87 70.85 2.33
13	16598	2627.9	286.88	-0.94 -0.09 -0.70 136.80 53.29 -15.67
14	16286	2362.3	71.45	-1.86 -0.99 -0.93 100.61 24.57 -2.24
15	16230	2515.9	317.29	2.18 -0.17 -1.89 103.36 70.04 -0.97
16	16112	3193.9	-90.52	-1.46 0.55 -0.85 92.62 85.45 32.38
17	16110	2232.9	140.98	0.81 -0.81 -1.12 135.66 38.59 1.04
18	16090	2405	-62.18	1.57 0.19 -0.86 136.95 39.46 22.59
19	16066	2431.4	97.62	1.59 -1.00 1.84 120.92 73.40 -3.70
20	16036	2851.3	-140.97	2.37 0.06 -2.95 102.15 106.92 25.62

Table S4. Docking results of TLR4- vaccine complex

Solution No	Score	Area	ACE	Transformation
1	19700	2868.5	396.86	1.63 0.80 1.53 -9.48 10.89 -1.98

2	19542	3035.8	362.04	-3.13 -0.08 1.77 38.93 5.17 -46.35
3	18860	2944.4	435.30	1.94 0.12 0.36 4.30 27.65 -25.29
4	18504	3117.7	-47.13	-0.91 0.39 -0.90 -64.10 22.25 -17.19
5	18198	3910.7	339.77	-0.48 0.21 -0.96 5.98 -16.30 -14.50
6	18094	2792.2	473.40	-74.55
7	18044	3527.8	446.36	0.87 0.92 -2.13 19.73 -5.61 -30.96
8	17882	3357.7	413.36	1.75 1.49 -2.58 -14.06 18.61 -4.49
9	17736	2196.3	448.36	-1.70 1.56 2.60 -88.08 3.34 -13.25
10	17682	2501.8	250.68	2.54 -0.07 0.52 38.86 -8.56 -42.13
11	17488	2060	425.03	2.89 -0.12 1.91 -66.45 -39.90 11.42
12	16950	2683.6	270.26	-0.04 -0.21 1.61 -67.56 13.68 -10.99
13	16874	3294.6	359.63	-0.02 1.04 1.66 0.03 7.79 -2.04
14	16838	2308	414.11	-0.08 -0.36 1.64 -77.49 1.19 -15.98
15	16824	2708.7	96.95	-2.98 0.05 2.18 30.86 14.48 -50.14
16	16638	2651	436.45	-2.99 -0.10 2.06 37.67 8.17 -48.55
17	16506	3000.7	480.74	2.54 0.75 -1.00 2.20 -26.37 -25.55
18	16498	2426.8	295.86	-0.01 1.00 -1.13 26.07 -31.93 -69.47
19	16468	2867	472.00	0.09 -0.42 1.60 -77.45 -1.77 -13.94
20	16466	2937.8	10.15	-1.04 0.50 -0.63 -59.40 19.93 -14.40

Table S5. Top 10 refine docked complex of MHC-I vaccine generated by fire dock server

Rank	Solution Number	Global Energy	Attractive VdW	Repulsive VdW	ACE	HB
1	10	0.00	0.00	0.00	0.00	0.00
2	7	5.71	-2.71	0.06	2.76	0.00
3	4	6.84	-2.88	2.57	0.16	-0.25
4	6	12.60	-22.20	3.19	12.91	-1.25
5	2	14.51	-0.25	0.00	0.40	-0.25
6	3	23.13	-22.01	20.49	12.45	-3.55
7	5	78.81	-35.39	140.14	10.83	-6.68
8	8	83.72	-28.77	119.19	14.51	-2.20
9	1	3160.71	-46.27	3963.28	16.85	-4.78

10	9	3722.01	-43.44	4733.49	6.01	-10.16

Table S6. Top 10 refine docked complex of MHC-II vaccine generated by fire dock server

Rank	Solution Number	Global Energy	Attractive VdW	Repulsive VdW	ACE	HB
1	9	4.53	-3.14	1.84	-0.29	0.00
2	6	8.63	-16.93	5.52	12.28	-1.57
3	4	28.59	-7.04	4.51	5.11	0.00
4	7	29.82	-1.64	0.00	0.51	-0.92
5	10	39.27	-28.21	57.47	-2.00	-1.80
6	8	347.59	-23.04	432.19	4.21	-0.70
7	5	1283.98	-21.02	1589.60	2.39	-0.77
8	2	1829.35	-21.70	2311.63	5.96	-0.83
9	1	3370.38	-37.96	4181.56	12.62	-2.68
10	3	7612.30	-70.28	9657.09	-15.33	-6.32

Table S7. Top 10 refine docked complex of TLR-4vaccine generated by fire dock server

Rank	Solution Number	Global Energy	Attractive VdW	Repulsive VdW	ACE	HB
1	6	-6.81	-9.76	1.81	8.31	-1.62
2	1	25.82	-45.71	38.20	19.00	-0.51
3	2	30.06	-14.23	4.26	10.19	-2.48
4	8	137.13	-24.94	159.97	8.68	-2.43
5	9	155.30	-27.60	199.94	13.93	-2.93
6	4	1061.39	-57.39	1448.80	-0.15	-8.41

7	10	1200.99	-24.61	1524.45	9.63	-5.39
8	7	1658.12	-66.38	2143.50	13.41	-5.96
9	3	2503.28	-67.38	3183.41	12.86	-9.62
10	5	3416.33	-44.98	4297.77	17.04	-7.07

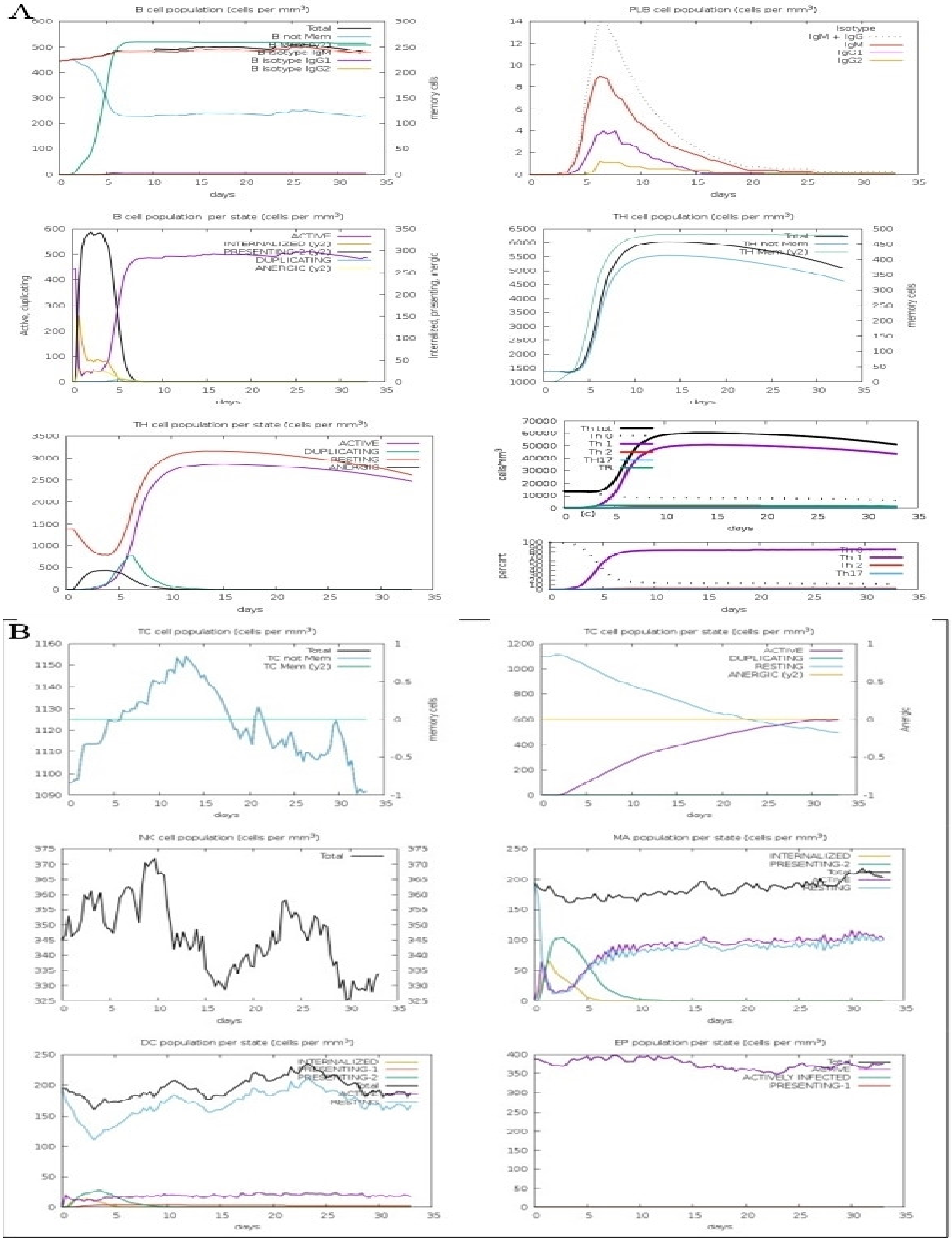

Figure S1. A. Different B-cell counts in response to the designed vaccine model. B. Different types of cellular immune response toward designed vaccine especially in the form of T-cell responses i.e. cytotoxic killer T-cells (Tc), natural killer cells (NK), dendritic cells (DC), macrophages (Mφ), and epithelial cells.
